# Supplementary material for: Causal interplay between lactose intolerance and gut microbiota: a combined bidirectional Mendelian randomization and in vivo validation study
Source: Front Nutr. 2026 Jun 1;13:1803337. doi: 10.3389/fnut.2026.1803337 (PMC13265576; doi:10.3389/fnut.2026.1803337)
Supplement: Supplementary file 1 [file Data_Sheet_1.zip › supplementary materials/Forward/forest plot/ebi-a-GCST90027680.finngen_R12_E4_LACTONAS.pdf]

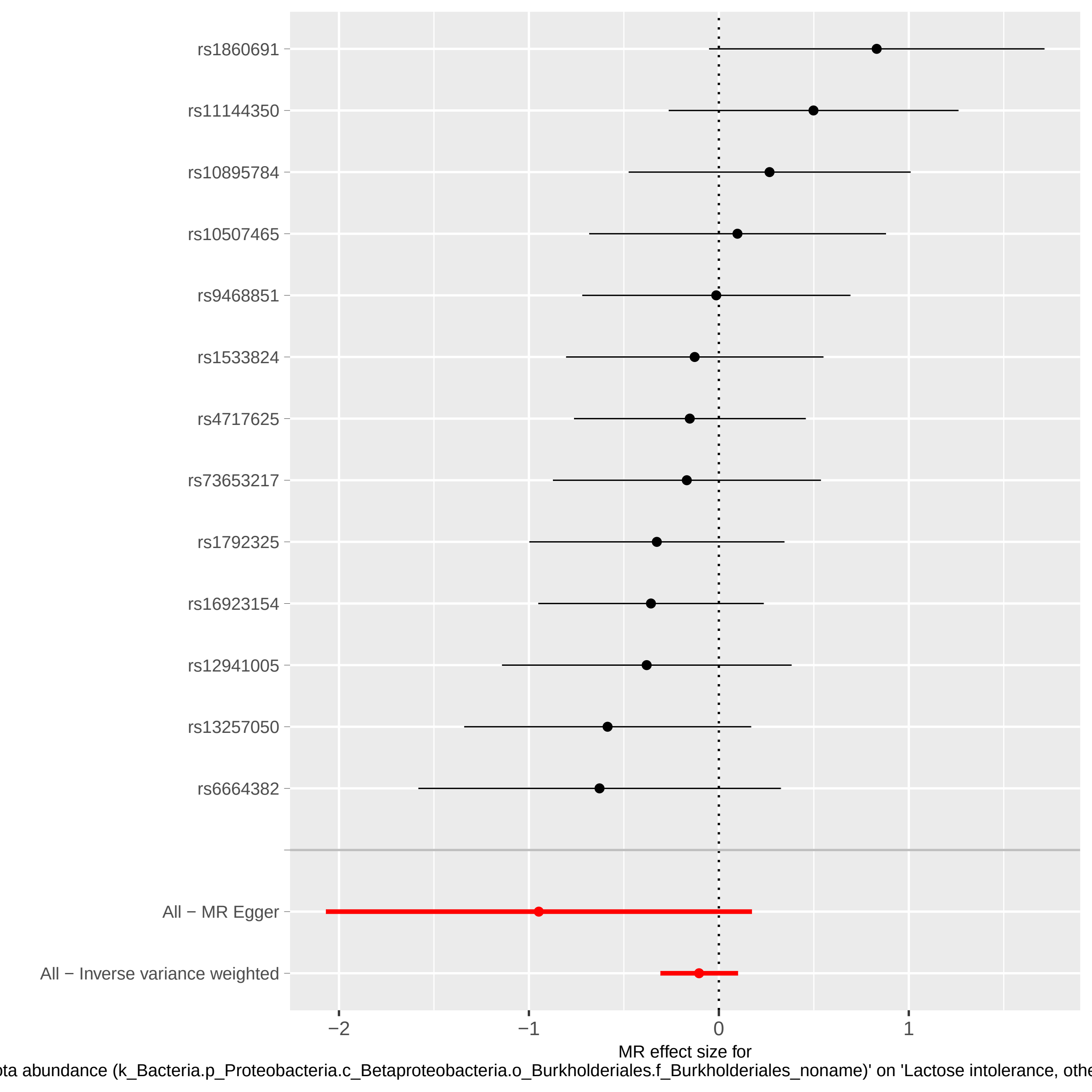

rs1860691
rs11144350
rs10895784
rs10507465
rs9468851
rs1533824
rs4717625
rs73653217
rs1792325
rs16923154
rs12941005
rs13257050
rs6664382
All − MR Egger
All − Inverse variance weighted
−2
−1
0
1
MR effect size for
ota abundance (k_Bacteria.p_Proteobacteria.c_Betaproteobacteria.o_Burkholderiales.f_Burkholderiales_noname)' on 'Lactose intolerance, othe
